# Quantitative Effects on Proximal Joints of Botulinum Toxin Treatment for Gastrocnemius Spasticity: A 4-Year-Old Case Study

**DOI:** 10.1155/2009/985717

**Published:** 2009-08-26

**Authors:** Veronica Cimolin, Manuela Galli, Marcello Crivellini, Giorgio Albertini

**Affiliations:** ^1^Bioengineering Department, Politecnico di Milano, Piaza Leonardo da Vinci 32, 20133 Milano, Italy; ^2^IRCCS “San Raffaele Pisana”, Tosinvest Sanità, Via della Pisana 235, 010163 Roma, Italy

## Abstract

Botulinum toxin A (BTA) is a recognized treatment for the early management of spasticity in children with Cerebral Palsy. This study quantified with Gait Analysis (GA) the gait pattern of a 4-year-old diplegic child with calf contracture before, 5 days, and 3 months after BTA injections into gastrocnemius. Kinematic and kinetic data of main lower limb joints were investigated. After only 5 days, ankle dorsi-plantarflexion and knee flex-extension improved, but hip joint worsened, increasing its excessive flexion, to compensate the improvement in knee position of the treated limb and to obtain better stability. A worsening of hip power happened. After 3 months, all joints generally improved their position during gait cycle. Hip and knee joints increased their range of movement and improvements occurred at ankle kinematics and kinetisc, too; a better ankle position and an increase of its capacity of propulsion during terminal stance were evident.

## 1. Introduction

The use of Botulinum toxin type A (BTA) has been recommended in the management of spasticity in ambulant children with Cerebral Palsy (CP) to improve function and to delay the development of fixed deformity and the need for surgical intervention. Although its immediate positive effects have been quantified using Gait Analysis (GA) in previous studies, especially for the treatment of calf contracture [1–8], some limits emerged. First, in literature the evaluation of gait changes after BTA injections for reducing gastrocnemius contracture was focused only on joints directly connected to gastrocnemius (knee and ankle joints); concerning proximal joints (pelvis and hip joints) no analyses were found. Then, all the studies quantifying both kinematic and kinetic strategies were conducted only on patients older than 6 years; few studies, in fact, investigated gait pattern of younger children using GA, and in these analyses only kinematics was evaluated, neglecting kinetic data [2, 8]. It is important to underline that no study evaluated the effects of the treatment few days after it, too; the evaluations were generally conducted one or more months after the treatment. 

 This study quantified with GA the effects of BTA injections into gastrocnemius for the reduction of excessive knee flexion due to calf spasticity in a 4-yr-old child with CP. His gait was evaluated considering distal and proximal joints and in term of kinematics, and kinetic data, too. He was examined before, 5 days, and 3 months after BTA injections into left gastrocnemius. In this way a complete quantification of motor strategy of a very young patient was conducted.

## 2. Case Presentation

The patient was a 4-yr-old male (weight: 16 Kg; height: 103 cm) affected by spastic diplegia; he was born approximately 7 weeks preterm with respiratory distress. He showed a dynamic calf contracture and a stiffness of adductor muscles; the left side was more worsened than the right one. He was an independent community ambulator without the use of assistive devices or orthoses. 

 The patient was injected 2 times, and in each inoculation BTA (Botox, Allergan, USA) was injected intramuscularly in the same muscles and with the same dosage: in the left medial gastrocnemius (20 U) and in the left lateral gastrocnemius (15 U). The temporal distance between the two inoculations was 10 months. No side effects or complications following injections were found. He was examined before (PRE session) and 5 days (POST1 session) after the first BTA injection and 3 months (POST2) after the second BTA injection. 

 The patient was evaluated with 3D GA, using a 12-camera optoelectronic system (ELITE2002, BTS S.p.A., Milan, Italy) with passive markers positioned according to Davis [9], for kinematic movement [10], two force platforms (Kistler, CH), for kinetic movement, and a synchronic video system (BTS S.p.A., Milan, Italy). Seven trials were collected for each session for the repeatability of the results. Some parameters were identified and calculated from kinematic and kinetic data: spatiotemporal parameters, angles joint values in specific gait cycle instant, and peak values of joint powers. The mean values (standard deviation) of kinematic and kinetic parameters are detailed, respectively, in Tables 1  and 2, in PRE, POSTs sessions, and for normative range (CG).

### 2.1. Pre Session

Child walked with a low velocity of progression (0.3 + 0.1 m/sec; CG: 1.2 + 0.2 m/sec) and limited anterior step length (right: 0.3 + 0.1 m and left: 0.3 + 0.2 m; CG: 0.4 + 0.1 m), if compared to normative values. Pelvic tilt was closed to normality. His gait was characterised by excessive left hip flexion at initial contact, while right side was closed to normality; a reduced range of motion was evident bilaterally, due to a limited extension in midstance. Knee was characterised by abnormal flexion during stance, due to spasticity of gastrocnemius, showing a limited range of motion bilaterally. Ankle joint was dorsiflexed during all gait cycle, and the right side was the most compromised. Right foot was in a correct position in the transversal plane, while left foot was intrarotated during all gait cycle. Concern kinetic data, a limited capacity of ankle propulsion during terminal stance and a reduced peak of hip power generation during stance phase were found bilaterally.

### 2.2. POST1 Session (5 Days After the First Injection)

Velocity of progression improved (0.7 ± 0.2 m/sec), while the other spatiotemporal parameters were unchanged. Pelvic tilt was in anterior position bilaterally and hip increased its flexion on right side, while left one improved its extension in midstance, remaining above normality. Right knee joint did not change significantly, while the left side improved its position in stance. The treatment reduced ankle dorsiflexion at right side while left one remained generally unchanged. There is a persistent drop foot in swing phase bilaterally, since ankle joints were not able to achieve dorsiflexion. Both feet were more intrarotated than PRE session. Concerning kinetic data, a reduction of power generation at left ankle during terminal stance occurred; hip power showed higher values of positive peak in stance phase bilaterally.

### 2.3. POST2 Session (3 Months After the Second Injection)

hip joint reduced its excessive flexion at initial contact on right side, remaining above normality, and improved its extension in mid-stance bilaterally, with consequently a better excursion during movement. Knee showed a more physiological position during stance phase with better range of motion. Ankle reduced its excessive dorsiflexion bilaterally. The right side was closed to normality during stance phase, and the left side showed, in addiction, the worsening of ankle dorsiflexion in swing phase. Foot progression improved bilaterally. Concern kinetics, hip power improved at right side, and maximum of ankle power increased bilaterally.

## 3. Discussion

In this study the kinematic and kinetic aspects of gait in a 4-yr-old child with CP were evaluated using GA to quantify the effects of BTA injection into gastrocnemius for the reduction of excessive knee flexion due to calf spasticity. Literature investigated widely this topic but it presents some limits: only the effects on ankle and knee pattern were evaluated and those on proximal joints, as hip and pelvic joints, were neglected. Then, patients were generally more than 6-yr-old; few studies investigated gait strategy in younger children using GA, but these analyses concerned only kinematics, not kinetic data. In addition, the evaluations were conducted generally one or more months after the treatment. 

 The results of this study demonstrated that the brief term effects of BTA occurred on all joints of lower limbs, not only on joints directly connected to treated gastrocnemius, knee, and ankle joints. Concerning pelvic joint, a worsening happened in the sagittal plane, increasing the anterior tilt; hip increased its flexion and this condition was directly connected to abnormal pelvic tilt position. The higher hip flexion at initial contact and the reduction of excessive knee flexion during stance phase on the treated side induced a new biomechanical position: this condition may be directly connected to the initial reduction of gastrocnemius spasticity that allows the subject to experience greater extension of left knee. At ankle joint some significant improvements occurred in the sagittal plane, while on coronal plane, the treatment caused a worsening in foot progression bilaterally. The excessive hip flexion and foot intrarotation may be a strategy to obtain a better stability and equilibrium. As knee joint has a more physiological pattern on the treated side, while the right side maintained its excessive flexion, this strategy probably is a scheme that permits to walk the patient with safety and at higher velocity of progression. In term of kinetics, before the treatment, the spastic gastrocnemius induced a reduction of ankle power generation at terminal stance, because this muscle is not able to strongly contract in this phase; after injection no changes occurred. This result was connected to the nature of BTA that denervates and produces a temporary paralysis of the treated muscle. The worsening of positive hip power in stance may be related to the high hip flexion at initial contact and to the reduced ankle power generation at terminal stance, too: the low levels of ankle work resulted in greater amounts of work being done by muscle groups of the hip. 

 Three months after the second injection of BTA, gait pattern revealed a general improvement, both at proximal and distal joints. Hip reduced its abnormal flexion at initial contact on right side and revealed an improvement in hip extension in mid-stance bilaterally. A better position and a good excursion of knee during all gait cycle bilaterally were evident. In this way a reduction of knee asymmetry, present in POST1 session, disappeared, allowing the subject to show more physiological knee flexion during most of gait cycle: a knee flexion reduction and a better extension would facilitate a corresponding increase of hip extension, to maintain erect posture during gait. Ankle improved its position during all stance phase; in swing phase instead the range of motion remained low. Even if the treatment reduced the dominance of the ankle plantarflexors over the antagonist muscles and so tibialis anterior is not contrasted by the abnormal gastrocnemius activity any more, probably the dorsiflexor muscle is again weak avoiding the correct position of ankle joint during swing. Concern kinetic results, a better capacity of propulsion at ankle joint is evident : the improvement may be due to the conclusion of temporary paralysis of treated muscle that now can contract during terminal stance. Hip power reduced on the right side its maximum in stance phase, according to decrease of hip flexion. 

 From these results, it is evident that BTA represents an encouraging therapy for the reduction of spasticity in CP subjects. In particular this study demonstrated that the treatment performed on a very young patient has significant effects immediately few days after the treatment. These modifications, that were worsening at proximal joints and improvements at distal ones, may be due to the search of a new control strategy immediately required after the reduction of gastrocnemius spasticity: the modifications at distal joints, directly connected to treatment of gastrocnemius, probably induce postural adjustments at proximal joints. Some months after the second inoculation, gait pattern revealed a general improvement, both at proximal and distal joints, demonstrating the reaching of better stability and reorganization of motor scheme of the patient. These significant changes may be directly connected to the effects of the BTA inoculation into the spastic muscles and to the natural maturation of the gait in the patient, too: the reduction of the spasticity during the growth period may reduce muscle tone and allow normal longitudinal muscle growth and lengthening leading to a more physiological gait pattern. Future studies might be conducted to compare children with the same age treated and not treated with BTA, in order to exclude one effect (the natural maturation of gait) with respect to the other (effects of BTA).

## Figures and Tables

**Table 1:** Summary of kinematic parameters (mean and standard deviation) for analysed patient (CP patient; right and left side) in the examined sessions and normative data (Control Group, CG). ROM: Range Of Motion; Min: Minimum value; Max: Maximum value; IC: Initial Contact; St: Stance phase; Sw: Swing phase.

	CP patient PRE		CP patient POST1 (5 days)		CP patient POST2 (3 months)		CG

	right	left	right	left	right	left	
Pelvic Tilt (degrees)							
* *Mean Pelvic Tilt	6.6 (1.3)	6.8 (0.4)	16.2 (1.7)	16.4 (2.1)	15.7 (0.8)	16.0 (0.1)	8.8 (4.3)
* *ROM	5.9 (1.5)	5.4 (0.3)	7.5 (2.1)	6.5 (1.1)	5.7 (0.5)	5.0 (1.5)	1.6 (3.6)
Hip Flex-Extension (degrees)							
* *IC	30.4 (0.1)	38.4 (1.4)	45.5 (0.7)	34.4 (0.9)	40.5 (0.5)	33.2 (1.5)	29.5 (4.4)
* *Min in St	6.4 (0.9)	21.5 (2.1)	13.8 (1.1)	16.0 (1.2)	−0.8 (2.5)	10.9 (0.3)	−8.7 (6.4)
Knee Flex-Extension (degrees)							
* *IC	29.3 (1.8)	35.7 (1.1)	25.8 (2.2)	22.3 (3.7)	16.4 (2.3)	13.6 (2.3)	6.7 (5.5)
* *Min in misSt	23.4 (3.8)	25.7 (1.0)	22.1 (0.4)	13.2 (3.9)	0.4 (2.7)	2.0 (0.9)	4.2 (2.1)
* *ROM	29.8 (3.9)	22.4 (1.2)	33.7 (0.8)	27.7 (2.6)	61.1 (2.7)	47.5 (1.7)	58.8 (4.7)
Ankle Dorsi-Plantarflexion (degrees)							
* *IC	14.4 (2.5)	9.3 (0.6)	9.4 (1.5)	10.4 (2.3)	−3.9 (1.3)	−3.0 (0.9)	−0.5 (4.8)
* *Max in St	29.4 (1.6)	18.7 (3.1)	19.7 (3.1)	24.4 (2.7)	14.7 (1.8)	8.7 (0.9)	12.2 (5.5)
* *ROM in Sw	8.3 (2.2)	4.3 (3.3)	4.6 (7.1)	8.1 (1.7)	5.4 (0.9)	4.2 (2.5)	23.3 (5.5)
Foot Progression (degrees)							
* *Mean foot progression	−13.2 (3.3)	7.3 (1.5)	3.2 (1.6)	21.4 (0.3)	−12.2 (0.2)	−3.4 (2.8)	−12.7 (4.4)

**Table 2:** Summary of kinetic parameters (mean and standard deviation) for analysed patient (CP patient; right and left side) in the examined sessions and normative data (Control Group, CG). Max: Maximum value; St: Stance phase.

	CP patient PRE		CP patient POST1 (5 days)		CP patient POST2 (3 months)		CG

	right	left	right	left	right	left	
Hip Power (W/Kg)							
Max of generation in St	0.2 (0.1)	0.3 (0.2)	0.8 (0.2)	0.5 (0.2)	0.5 (0.3)	0.4 (0.4)	0.8 (0.4)
Ankle Power (W/Kg)							
Max of generation during terminal St	0.7 (0.5)	1.0 (0.9)	0.6 (0.2)	0.4 (0.3)	2.7 (0.1)	2.6 (0.7)	3.3 (1.1)
